# New Insight into the Systematics of European *Lepidocyrtus* (Collembola: Entomobryidae) Using Molecular and Morphological Data

**DOI:** 10.3390/insects11050302

**Published:** 2020-05-13

**Authors:** Daniel Winkler, Eduardo Mateos, György Traser, Ferenc Lakatos, Viktória Tóth

**Affiliations:** 1 Institute of Wildlife Management and Vertebrate Zoology, Faculty of Forestry, University of Sopron, Bajcsy-Zsilinszky u. 4, H–9400 Sopron, Hungary; winklerdanielandras@gmail.com; 2 Departament de Biologia Evolutiva, Ecologia i Ciències Ambientals, Facultat de Biologia, Universitat de Barcelona, Avinguda Diagonal 643, 08028 Barcelona, Spain; emateos@ub.edu; 3 Institute of Silviculture and Forest Protection, Faculty of Forestry, University of Sopron, Bajcsy-Zsilinszky u. 4, H–9400 Sopron, Hungary; tgyn49@gmail.com (G.T.); toth.viktoria@uni-sopron.hu (V.T.)

**Keywords:** subgeneric division, Ascocyrtus, *Cinctocyrtus*, *Lanocyrtus*, *Setogaster*, COII, EF-1α, phylogeny, taxonomy, cryptic species

## Abstract

The Collembolan genus *Lepidocyrtus* is subdivided into up to eight subgenera, of which only *Lepidocyrtus* s.str. (Bourlet, 1839) and *Lanocyrtus* (Yoshii & Suhardjono, 1989) are represented by European species. The discovery of unique characters in the European species *Lepidocyrtus tomosvaryi* (rounded dental tubercle) and *L. peisonis* (lateral tuft of long filiform chaetae in abdomen III) has only described so far for species of the subgenera *Setogaster* (Salmon, 1951) and *Cinctocyrtus* (Yoshii & Suhardjono, 1989) and has raised the need to perform a molecular analysis by involving other representative species of the genus. For this study, phylogenetic analysis of 15 *Lepidocyrtus* species occurring in the Carpathian Basin were carried out. The analyses, which was based on both concatenated datasets of COII and EF1-α sequences and individual gene sequences, clearly placed *L. tomosvaryi* within the subgenus *Lanocyrtus* and *L. peisonis* within *Lepidocyrtus* s.srt. European species groups defined on the basis of morphological characters were only partly confirmed by the concatenated and COII analyses because of the splitting of the *pallidus–serbicus*-group, whereas EF1- α sequences weakly supported this group.

## 1. Introduction

*Lepidocyrtus* (Bourlet, 1839) is considered to be among the most problematic collembolan taxa [1,2]. With 233 species currently reported globally [3], *Lepidocyrtus* is one of the most species-rich and widespread genera, inhabiting a wide range of habitats from xerophilous areas (e.g., [4,5]) through caves (e.g., [6,7]) to forests (e.g., [8,9]) and wetlands [10]. To date, a total of 35 *Lepidocyrtus* species have been described in Europe [3], albeit the real number is likely higher considering the probable presence of cryptic species as suggested by several authors [11,12,13,14,15]. The main characteristic features of the genus include 8 + 8 eyes, four-segment antennae, body and ventral surface of furcula with scales, and mucro bidentate [16]. After the essential works by Gisin [17,18,19,20] and Szeptycki [21,22], accurate information on dorsal cephalic and trunk chaetotaxy became inevitable in the diagnosis of species and species groups. Based on the abovementioned characteristics and the distribution of scales on various parts of the body, five species groups have been defined for European *Lepidocyrtus*: The *lusitanicus*-, *lignorum*-, *lanuginosus*-, *curvicollis*-, and *pallidus–serbicus*-groups, respectively [19,23,24,25,26,27,28].

Nevertheless, the taxonomy of *Lepidocyrtus* is rather complex and, on the worldwide level, the genus has been subdivided into several subgenera [9,29,30,31,32,33], of which diagnostic characters and their applicability have often been discussed by different authors [34,35,36]. Of the total of eight currently recognized subgenera (*sensu* Cipola [9]), only *Lepidocyrtus* s.str. (Bourlet, 1839) and *Lanocyrtus* (Yoshii & Suhardjono, 1989) are represented in the European fauna [13,37]. Both mentioned subgenera are characterized by the absence of a specific morphological feature, the dental tubercle at the basal part of the dens on the dorsal surface, while species belonging to the remaining subgenera possess this morphological character [32]. The vast majority of species with dental tubercle is distributed in the tropical and subtropical regions of East Asia [36].

Surprisingly enough, from Central Europe (Hungary), (Winkler and Traser) [27] described a species, namely *L. tomosvaryi* (Winkler & Traser, 2012), having a rounded dental tubercle, a character virtually unknown in any other European *Lepidocyrtus* species. Based on the subgenera division by Yoshii & Suhardjono [32], this species should apparently be classified into the subgenus *Cinctocyrtus* (Yoshii & Suhardjono, 1989) within the Ascocyrtus group because of the presence of the rounded dental tubercle and the absence of scales on antennae and legs. Nevertheless, its position among the European *Lepidocyrtus* species has remained uncertain and unclarified. Further curiosity was discovered while examining *L. peisonis* (Traser & Christian, 1992), another species originally described from Hungary. On the occasion of its revision and redescription, Winkler and Mateos [38] found a special feature (tuft of filiform chaetae on abdomen III in lateral position) not formerly explored in any European *Lepidocyrtus* species and which, so far, has only been detected in species of the subgenera *Setogaster* Salmon, 1951 and *Cinctocyrtus* [32,38,39]. These two characters (dental tubercle and tuft of long filiform chaetae), observed in the mentioned two species and considered to be unique among the European *Lepidocyrtus*, evoked a necessity to perform a molecular analysis by involving also other representative species of the genus.

Prior to the present study, Mateos et al. [13] carried out an extensive phylogenetic analysis on *Lepidocyrtus*, including 19 species from different European localities in order to prove, *inter alia*, the phylogenetic support of the 5 species groups defined using morphological characters.

The main aims of our study were to (i) reveal the position of the species *L. tomosvaryi* and *L. peisonis* with unique morphological features (rounded dental tubercle and lateral tuft of long filaments on abdomen III, respectively) among the European *Lepidocyrtus*, (ii) clarify the validity of dental tubercle in subgeneric division with respect to the European species, and (iii) ascertain whether the main European species-groups of *Lepidocyrtus* are supported by our molecular data. In this study, a total of 15 species were included, 8 of which were not present in the work of Mateos et al. [13] on European *Lepidocyrtus*.

## 2. Materials and Methods

### 2.1. Species Sampling and Processing

From a total of 15 *Lepidocyrtus* species extracted from samples collected in various habitats (cave, forest, meadows, wetlands) and locations in the Carpathian Basin (Hungary), 77 specimens were studied (Table 1; for specimen codes see Table S1). Specimen collection was partly carried out from surface of soil or stone, lying dead wood, and moss on tree barks using a hand-operated aspirator. Additionally, soil, litter, and moss samples were also collected, from which springtails were extracted using a modified Berlese-Tullgren apparatus without light or heating devices. In case of both methods, specimens were placed in plastic micro tubes filled with 96% ethanol and tagged with a specific sample code. In the case of four species (*L. florae* Winkler & Mateos, 2018, *L. isabelleae* Winkler 2017, *L. tomosvaryi*, and *L. traseri* Winkler 2016), specimens of the type material series were used for the molecular analyses, while specimens of further three species (*L. arrabonicus* (Traser, 2000)*, L. mariani* (Traser & Dányi, 2008) and *L. peisonis*) were collected from the exact type localities.

For morphological analysis, some specimens of each sample were cleared in Nesbitt’s solution and mounted in Hoyer medium on glass slides, labeled with the same code of samples in ethanol. The slides were then observed under a Leica DM2500 LED microscope with conventional bright light and phase contrast. The identification of species was performed using relevant keys and descriptions available [4,24,25,26,28,37,40]. Prior to DNA extraction, to ensure identity, specimens selected for molecular analysis were also examined in ethanol for major chaetotaxic characters using a modular Leica M205c stereomicroscope up to 160x magnification.

Of the five European *Lepidocyrtus* species groups, only four, namely the *lignorum*-, *lanuginosus*-, *curvicollis*-, and *pallidus–serbicus*-groups, are represented by the species collected and examined in this study (Table 2), since species belonging to the *lusitanicus*-group are only known from the Iberian Peninsula, Balearic Islands [41] and one locality in the French Pyrenees [42] so far.

The chaetotaxic nomenclature used throughout this paper follows the AMS nomenclature system [35] for dorsal cephalic macrochaetae, and Szeptycki [22] for dorsal schemes of thoracic and abdominal segments.

The following general morphological abbreviations are used: Ant.: Antennal segment; Th.: Thoracic segment; Abd: Abdominal segment; I-VI: Segment numbers; pse: Pseudopores.

### 2.2. Molecular Methods

#### 2.2.1. DNA Extraction and Sequencing

All samples were stored in 96% ethanol at 4 °C until DNA extraction. Voucher specimens and extracted DNA samples were stored at the institute’s collection. DNA was extracted from entire bodies using Thermo Scientific Phire Animal Tissue Direct PCR Kit following the manufacturer’s protocol. Eluted DNA was stored at −20 °C.

A segment of the mitochondrial gene cytochrome oxidase subunit II (COII) was amplified using tRNA-20-LcuJ (5′-GGTTTAAGAGACCGTGGCTTAC-3′) and tRNA-13-LcuN (5′-TCTAACGTGGCAGACTAGTGC-3′), as well as two additional primers of tRNA-K-LcuJ (5′-GAGCGTATTATAAAGCGGTTTAAG-3′) and tRNA-L-LcuN (5′-CAGACTAGTGCCATGAATTTAAGC-3′) (11). A fragment of the fragment of the elongation factor-1 alpha (EF1-α) gene was amplified using EFLcuJ (5′-ATGGGGGCAAGATAGCGTCAA-3′) and EFLcuN (5′-TGAAGGCTGAACGTGAACGTGG -3′) primers [11]. PCR amplifications were performed with Thermo Scientific Phire Hot Start II DNA Polymerase. Thermocycling consisted of an initial denaturation step at 98 °C for 5 min, followed by 40 cycles at 98 °C for 5 s, 48 °C for 5 s, and 72 °C for 20 s with a final extension step that lasted 1 min at 72 °C for the COII gene; 5 min at 98 °C followed by 40 cycles at 98 °C for 5 s, 55 °C for 5 s, and 72 °C for 20 s with a final extension step that lasted 1 min at 72 °C for the EF1-α gen.

Sequences were generated at the Eurofin’s Laboratory (Ebersberg, Germany). All sequences are available at NCBI GenBank (accession numbers: MT136169–MT136241, and MT153249–MT153288, Table S1).

#### 2.2.2. Data Analyses

For nuclear DNA (EF1-α) analyses, 39 individuals were used, and 72 individuals were entered to the mitochondrial DNA (COII) analyses (Table S1). Sequences were visualized using Sequence Scanner and ambiguous positions were corrected by hand. Subsequently, sequences were aligned using ClustalX [43]. Sequences of COII and EF1-α gene fragments were then concatenated, resulting in a 1061-bp-long final alignment.

Intra- and interspecific divergences of COII and EF1-α were calculated based on K2P [44] and uncorrected p-distances using MEGA 5.02. [45].

#### 2.2.3. Phylogenetic Analyses

As outgroups for the phylogenetic analyses, we used *Cyphoderus* gr. *bidenticulati* sensu Delamare-Deboutteville [46] data by Mateos et al. [13] taken from the GenBank (MF095527, MF095613) and our own sequences of *Orchesella cincta* (Linnaeus, 1758). We applied jModeltest 2.1.2 [47,48] to select the best model of nucleotide substitution with Akaike Information Criterion (AIC) [49]. The selected model was HKY+I+G for COII, while GTR+I+G was the best model for EF1-α. In the concatenated alignment, all three coding positions of the COII and EF1-α were included in the analyses, and the best-fitting substitution models were applied for each gene (partition). Maximum likelihood (ML) analysis was performed with MEGA 5.02. [45] in case of the COII and EF1-α dataset. The level of support for individual nodes was evaluated by bootstrapping with 5000 replicates. On the concatenated data set, the maximum likelihood analyses were performed using the software IQ-TREE 1.6.12 [50]. Node support was assessed by 1000 bootstrap replicates.

Bayesian Inference analyses were performed by MrBayes v. 3.2. [51]. A stop rule was applied during the run when the value reached 0.01, which occurred on the 57,900,000 (COII), 8,610,000 (EF1-α), and 7,398,000 (concatenated) Markov Chain Monte Carlo (MCMC) generations with two chains. MCMC started from a random tree, sampling one tree every 10,000 generations. The first 25% of the trees were discarded as a burn-in. To test convergence, the software Tracer v1.7 [52] was used. Effective sample size (ESS) values exceeded 200 for all estimated parameters. Trees from Bayesian analyses were presented using FigTree v. 1.4.4. [53].

## 3. Results

### Molecular Analyses

A total of 72 sequences were obtained for the mitochondrial COII gene with a total length of 552 bp for all 15 species (Table S1). For the nuclear gene EF1-α, 39 sequences were gained from 14 species (omitting *L. arrabonicus*) containing 80 bp of the first exon, 88–124 bp of the intron, and 279 bp of the second exon. Interspecific uncorrected p-distances based on COII (Table S2) ranged from 14.5% (between *L. nigrescens* Szeptycki, 1967 and *L. paradoxus* Uzel, 1890) to 31.0% (between *L. curvicollis* Bourlet, 1839 and *L. isabelleae*). Extreme values of mean K2P distances (Table S3) were observed between the same species pairs, ranging from 16.4% (between *L. nigrescens* and *L. paradoxus*) to 40.8% (between *L. curvicollis* and *L. isabelleae*).

**Figure 1:**
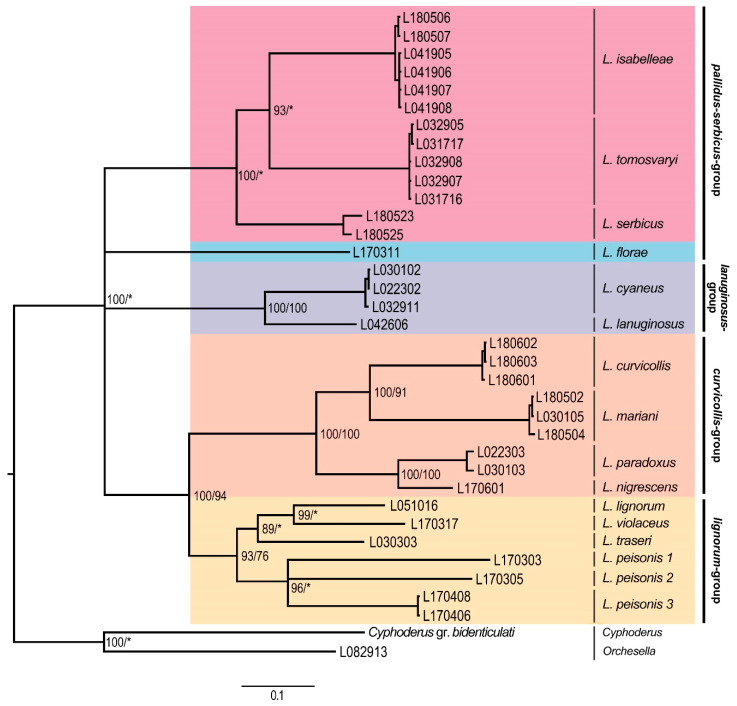
Bayesian consensus tree based on the concatenated (COII and EF1-α) dataset. Numbers close to nodes denote Bayesian posterior probabilities (BI)/maximum likelihood (ML) bootstrap support. Asterisks (*) indicate posterior probabilities or bootstrap values less than 60%. Morphological groups are labeled in bold.

**Figure 2:**
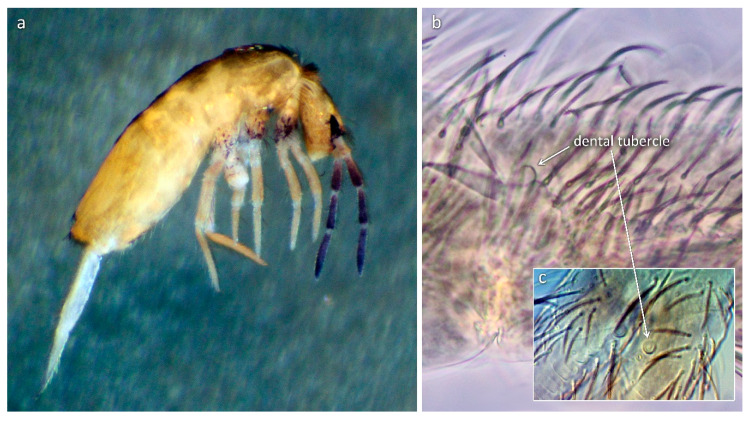
*Lepidocyrtus tomosvaryi*: (**a**) Habitus; basal part of dens with rounded dental tubercle in (**b**) lateral and (**c**) dorsal view.

The concatenated phylogenetic tree constructed from the COII and EF1-α genes presented five major clades (Figure 1), only partly corresponding with the morphological species groups as defined in Table 2 because of the splitting of the *pallidus–serbicus*-group, which appeared as a polytomy of two clades in the upper position of the tree. The species from *pallidus–serbicus*-group bearing cephalic macrochaetae A_0_A_2_A_3_M_2_S_3_Pa_5_ and with body macrochaetae formula 00/0101+2, namely *L. isabelleae*, *L. serbicus*, and the only European species with dental tubercle, *L. tomosvaryi* (Figure 2a-c), were clustered as sister taxa forming a monophyletic group strongly supported by Bayesian posterior probability (100%). *L. florae*, a species also belonging to the *pallidus–serbicus*-group but omitting head macrochaeta M_2_, appeared as sister clade.

The *lanuginosus*-group, including the species *L. cyaneus* (Tullberg, 1871), and *L. lanuginosus* (Gmelin, 1788) and characterized by body macrochaetae formula 10/0101+2, formed a separate clad maximally supported by both BI and ML.

**Figure 3:**
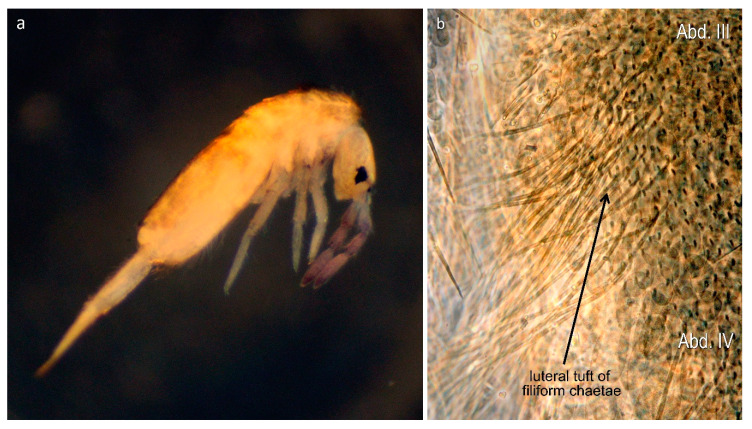
*Lepidocyrtus peisonis:* (**a**) Habitus; (**b**) tuft of filiform chaetae on the lateral part of Abd. III.

The fourth highly supported clade was formed by the *curvicollis* and *lignorum*-groups. A strong support (100% for both BI and ML, respectively) for the subclade representing the species of the *curvicollis*-group, namely *L. curvicollis*, *L. mariani*, *L. nigrescens,* and *L. paradoxus*, was found. Concerning the *lignorum*-group, the BI method yielded a strong support (93%), while the ML method provided lower (76%) assignment success for this clade, composed of the species *L. lignorum* (Fabricius, 1793), *L. violaceus* (Geoffroy, 1762), *L. traseri*, and *L. peisonis* (Figure 3a). Within this clade, populations of the latter species, bearing a lateral tuft of long filiform chaetae (Figure 3b), formed different monophyletic subgroups (*L. peisonis* 1, *L. peisonis* 2, and *L. peisonis* 3 in Figure 1), also suggesting the presence of cryptic species.

**Figure 4:**
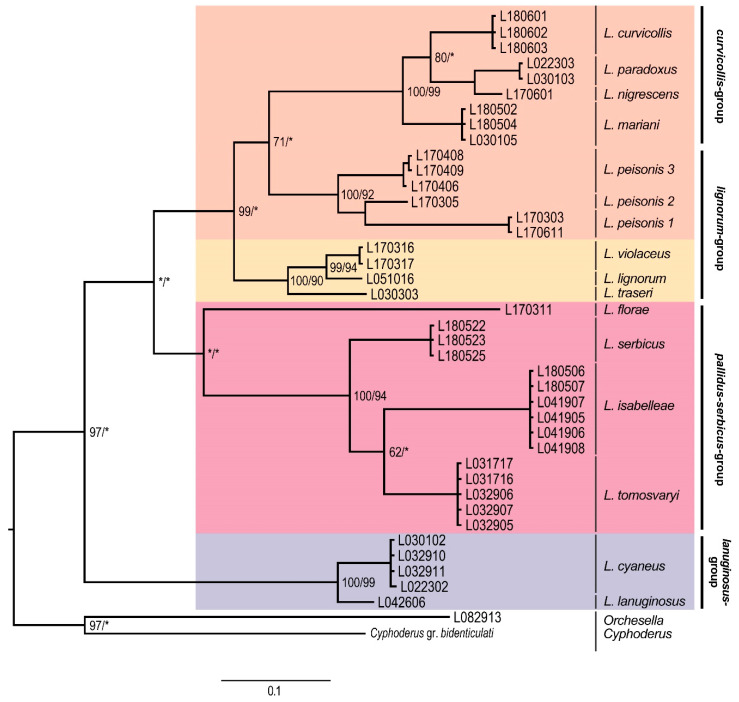
Bayesian consensus tree obtained based on the EF1-α dataset. Numbers close to nodes denote Bayesian posterior probabilities/maximum likelihood bootstrap support. Asterisks (*) indicate posterior probabilities or bootstrap values less than 60%. Morphological groups are labeled in bold.

Phylogenetic trees of individual genes show slightly different topologies with respect to the topology of the concatenated tree. With reference to the EF1-α phylogenetic tree (Figure 4), the most basal clade corresponded to the *lanuginosus*-group, forming a highly supported clade. The *pallidus–serbicus*-group, including *L. florae, L. serbicus, L. isabelleae*, and *L. tomosvaryi*, formed a monophyletic but only weakly supported group, as both ML and BI were below 60%. For the *curvicollis+lignorum* clade, only BI provided strong support. Within this clade, the species of *curvicollis*-group formed a monophyletic entity with high support, while species of *lignorum*-group split in two groups. On the one hand, specimens of the *L. peisonis* populations appeared as a sister clade to the *curvicollis*-group, despite their morphologic differences (in *L. peisonis*, Th.II only slightly projected, head macrochaeta A3 is present, while Abd. IV accessory chaeta s is absent, see Table 2). However, only BI yielded decent support (71%) for this topology. On the other hand, the other species of *lignorum*-group (namely *L. lignorum*, *L. traseri* and *L. violaceus*) appeared as highly supported clade and formed the sister group of the clade formed by the *L. peisonis* + *curvicollis*-group.

**Figure 5:**
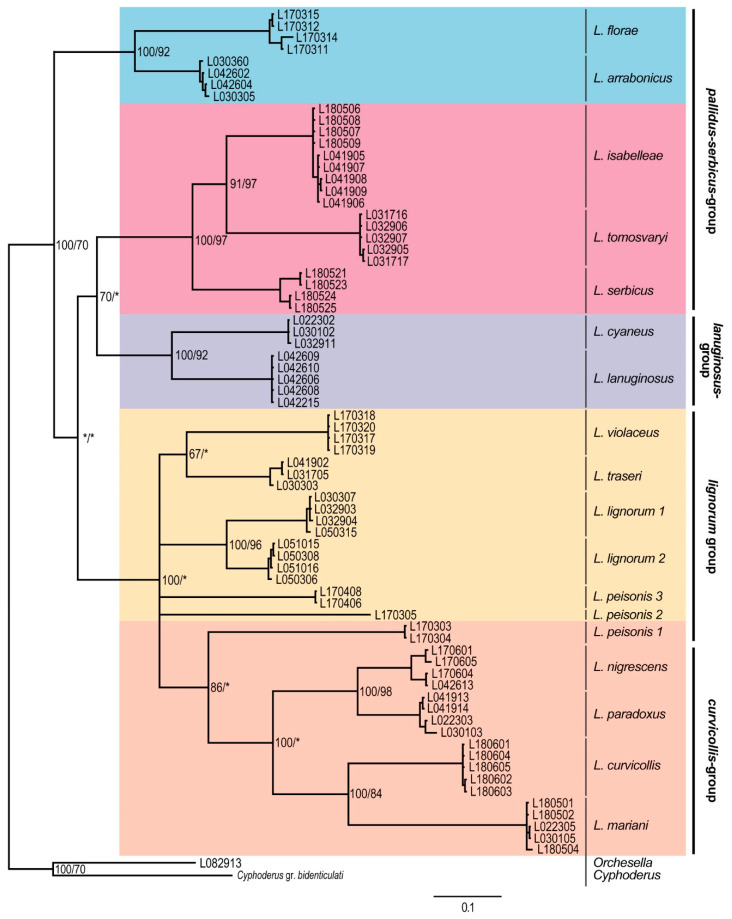
Bayesian consensus tree obtained based on the COII dataset. Numbers close to nodes denote Bayesian posterior probabilities/maximum likelihood bootstrap support. Asterisks (*) indicate posterior probabilities or bootstrap values less than 60%. Morphological groups are labeled in bold.

In the COII dataset (Figure 5), the species from the *pallidus–serbicus*-group were split in two groups. On the one hand, *L. arrabonicus* and *L. florae* formed a well-supported clade in the upmost position of the tree, while, on the other hand, *L. isabelleae*, *L. serbicus* and *L. tomosvaryi* formed a well-supported clade located as a sister group of the *lanuginosus*-group. The *curvicollis+lignorum* groups formed a clade highly supported by BI. Within this clade, the *curvicollis*-group appeared as a strongly supported (BI) monophyletic group, while species from the *lignorum*-group split in several branches. This splitting suggests the presence of cryptic species among the different sampled populations of both *L. lignorum* and *L. peisonis*, which is also emphasized by the high mean p-distance and K2P values (14.7 and 16.7 for *L. lignorum*, and 25.2–26.6 and 31.0–34.0 for *L. peisonis*, respectively) (Tables S2 and S3).

## 4. Discussion

The Carpathian Basin is known as one of the areas of highest biodiversity in Europe, owing to the different biogeographic influences [54], which is also well reflected in the rich Collembola fauna [55], including the relatively high number (18) of *Lepidocyrtus* species reported from Hungary [28]. Among the species occurring in the country, particular attention has been focused to those species (*L. tomosvaryi* and *L. peisonis*) bearing specific characters mostly typical for *Lepidocyrtus* inhabiting the tropical and subtropical regions of East Asia, America, or Australia [36,39]. Our molecular study with the supplementary morphological observations could shed light on the identity of these two species, the validity of subgeneric classification within the European *Lepidocyrtus*, and the conformity of species groups previously established on the basis of morphological characters [23,24,25,26,27,28].

### 4.1. Subgeneric Aspects of the European Lepidocyrtus

Based mostly on Oriental species, the first subgeneric classification system of the genus *Lepidocyrtus* was established by Yosii [30], which was subsequently revised and refined [1,9,31,32]. The fundamental determining character in Yoshii’s classification is the presence or absence and the shape of the basal dental tubercle. The only two subgenera lacking this feature, which is the subgenera to which all European species belong, are *Lepidocyrtus* s.str. (Bourlet, 1839) and *Lanocyrtus* (Yoshii & Suhardjono, 1989). The only separating character between these two subgenera is the presence of scales on the antennae, legs beyond coxae, and posterior face of manubrium in *Lepidocyrtus* s.str. and their absence in *Lanocyrtus* [32]. The species *L. tomosvaryi* would typically belong to the latter group. However, a marked difference, namely the presence of the dental tubercle, has already raised confusion in assigning the species to a particular subgenus [27]. Due to the presence of a rounded dental tubercle, *L. tomosvaryi* should apparently be placed in the *Ascocyrtus* group, more precisely in the subgenus *Cinctocyrtus* (Yoshii & Suhardjono, 1989) because of the absence of scales on antennae and femur [32,36]. Based on our molecular analyses, EF1-α sequence data clearly placed *L. tomosvaryi* among the *pallidus–serbicus* group and COII data within the *serbicus*-group *sensu* (Winkler) [56]. Accordingly, notwithstanding the presence of the dental tubercle, this species has close relation with those lacking this feature but sharing the same body macrochaetotaxy (00/0101+2) and distribution of scales, thus belonging to the subgenus *Lanocyrtus*.

According to our current knowledge, species possessing dental tubercle are mostly distributed in the tropical and subtropical regions of East and Southeast Asia, America, and Australia [1,35,39,57,58], whereas they rarely occur in the temperate zone [36]. This fact raises the possibility that the presence of the dental tubercle, apart from the geographic explanation, is of climatic origin [36]. Nevertheless, this assumption was weakened by the occurrence of a species possessing this feature in the temperate continental region (Central Europe). Wang et al. [36] drew attention to a morphological difference of dental tubercle between the Oriental species, where the tubercle is usually distinct with a clear basal socket, and species outside the Asian region, where this papilla is generally less clear from a barely observable hump to a clear projection, but almost never having any sort of basal socket. *L. tomosvaryi*, however, does not fall into the latter category, since a clear basal socket of the dental tubercle is easily observable (Figure 2c).

Whereas the taxonomic relevance of the basal dental tubercle as subgeneric diagnostic character has been proven, its validity and applicability on worldwide level has been often questioned [34,36]. Based on a phylogenetic analysis of Hawaiian *Lepidocyrtus*, the basal dental tubercle was found to be less determinative in delineating monophyletic lineages compared with other features, e.g., apical bulb on Ant. IV [34]. As a result of a phylogenetic analysis of *Lepidocyrtus* from Puerto Rico, the dental tubercle appeared to be one of the features with relatively high retention index, and thus appropriate as subgeneric diagnostic character for Neotropical species, but mostly combined with other characters [35]. Since the abovementioned studies are relevant to confined geographical regions only, several authors have advocated the need for extended molecular analyses to help discriminate genera of Lepidocyrtinae (e.g., [34,35,56]). In the original description, based on the chaetotaxic similarity with other European species, the authors decided to place *L. tomosvaryi* in the subgenus *Lanocyrtus* [27], which is strongly supported by the present molecular analyses.

A further character unique among European *Lepidocyrtus*, a tuft of numerous (up to 60) long filiform chaetae on the lateral part of Abd. III, was discovered in *L. peisonis* when performing a detailed redescription by Winkler and Mateos [40]. This character has been described for certain species belonging to the subgenera *Setogaster* and *Cinctocyrtus*, but it was virtually unknown for European species of the subgenera *Lanocyrtus* and *Lepidocyrtus* s.str. With scales on the antennae, legs and dorsal surface of manubrium, the species *L. peisonis* belongs to the subgenus *Lepidocyrtus* s.str. In contrast, both *Cinctocyrtus* and *Setogaster* are characterized by lack of scales on the abovementioned regions and the presence of rounded dental tubercle [32,39]. An additional important character for *Setogaster* is the presence of an accessorial spinelet on the basal mucronal spine [9,39,59]. Our molecular data clearly placed *L. peisonis* in the subgenus *Lepidocyrtus* s.str., within the *lignorum*-group, showing that the lateral tuft alone is not regarded as a decisive character at subgeneric level and should be taken into account in combination with other characters.

Corroborating with the result of Mateos et al. [13], the 15 European species examined in our study can be classified either within the subgenus *Lepidocyrtus* s.str. or *Lanocyrtus*. Our molecular analyses also confirmed the monophyly of the subgenus *Lepidocyrtus* s.str. and the paraphyly of *Lanocyrtus*.

### 4.2. Lepidocyrtus Species Groups

The molecular analyses gave us the opportunity to confirm the validity of the major *Lepidocyrtus* species-groups created on the base of morphological characters (see the key in [13], p. 649). The *lanuginosus*-group, represented by *L. cyaneus* and *L. lanuginosus* in our study, was strongly supported by the genetic datasets, supporting the results obtained by Mateos et al. [13]. With respect to the *pallidus–serbicus*-group, its monophyly has only been suggested by the EF1-α dataset. However, it was not sufficiently supported. COII and concatenated (COII and EF1-α) datasets classified the species bearing cephalic macrochaetae A_0_A_2_A_3_M_2_S_3_Pa_5_ and with body macrochaetae formula 00/0101+2 (*L. isabelleae, L. serbicus* and *L. tomosvaryi*) into a strongly supported monophyletic clade, while the species *L. arrabonicus* and *L. florae*, having the same body macrochaetae formula but reduced number of head macrochaetae (A_0_A_2_A_3_Pa_5_ and A_0_A_2_A_3_S_3_Pa_5_, respectively), were clustered in a highly supported separated clade in the COII tree (in the concatenated tree, a single sequence of *L. florae* represented a separate clade, since EF1-α sequences of *L. arrabonicus* were unfortunately not available).

Nevertheless, dorsal head macrochaetotaxy can be variable within species groups, as a recently described species, *L. intermedius* (Mateos, Escuer & Álvarez-Presas, 2018), with dorsal head macrochaetae A_0_A_2_Pa_5_ typical for the *curvicollis*-group, was clearly placed within the *lignorum*-group in the phylogenetic analysis carried out by Mateos et al. [13]. It can therefore be stated that dorsal head macrochaetotaxy is not suitable for species-group differentiation, and for this very reason, extended analyses involving more species and more genes will probably verify the validity of the *pallidus–serbicus*-group as well. In the concatenated dataset, the strongly supported *curvicollis*-group, appearing as a sister clade with the *lignorum*-group, is further divided into two subclades. The connection between the species *L. curvicollis* and *L. mariani* might be derived from the presence of additional pseudopores on Abd. IV: in dorsolateral (*L. curvicollis*) and lateral position (*L. mariani*), which supports the suggestion by Deharveng et al. [60], according to which the number and distribution of pseudopores may have high taxonomic importance.

Within the *lignorum*-group, based on the concatenated datasets, specimens of the two sampled *L. peisonis* populations are grouped in a separate subclade. Apart from the lateral tuft of filiform chaetae, the other unique character among the studied species is the truncated unguiculus of this species (see Table 2). The shape of the unguiculus was one of the phylogenetically important characters in Soto-Adames’ analyses of Neotropical *Lepidocyrtus* [35]. Whether the separation of *L. peisonis* within the *lignorum*-group is derived from either of the abovementioned characters remains an open question.

### 4.3. Cryptic Species

As several authors have already pointed out, the species richness of *Lepidocyrtus* is most likely highly underestimated due to the presence of cryptic species that cannot be delimited and diagnosed using traditional morphological characters [11,12,13,14,15,61]. Based on our molecular datasets, several cryptic species were detected in the *lignorum*-group. In the COII tree, specimens determined as *L. lignorum* were classified in two different clades. At the same time, specimens identified as *L. peisonis* appeared separately in three different clades in all phylogenetic trees. The obtained K2P and p-distance values (Tables S2 and S3) were way above the mean intraspecific values reported by Zhang et al. (2018) and Mateos et al. (2018) for the species of the genus (0%–7.0% for K2P and 0%–6.5% for p-distance, respectively), confirming cryptic species status. Nevertheless, consideration should be given to the revision of rarely considered or overlooked characters for these species, including, e.g., the number, type, and distribution of S-chaetae [62], or the number and distribution of pseudopores [60].

## 5. Conclusions

The present molecular study brought new insight into the phylogeny of European *Lepidocyrtus*. One of the key characters in delimiting subgenera, the dental tubercle, has been proven to be not determinative in delineating monophyletic lineages. Based on the morphological pecularities, *L. tomosvaryi*, the only European species possessing a dental tubercle, would more likely belong to the *Cinctocyrtus* subgenus. Nevertheless, our molecular analyses clearly placed this species within a clade composed of species lacking this feature but sharing the same body macrochaetotaxy and distribution of scales, thus belonging to the subgenus *Lanocyrtus*.

The molecular phylogeny confirmed the monophyly of the subgenus *Lepidocyrtus* s.str. and the paraphyly of *Lanocyrtus*. European species groups established for the genus on the basis of morphological characters were only confirmed in part. The splitting of the *pallidus–serbicus*-group in the COII and concatenated trees requires further analysis involving extended material and genes to clarify the validity of this group.

## Figures and Tables

**Table 1:** Collection and locality data of the *Lepidocyrtus* material examined.

Genus	Species	Nr of Spec.	Municipality	Habitat	Position (WGS 84)	Date of Collection
*Lepidocyrtus*	*arrabonicus*	4	Fertőújlak (loc. typ.)	meadow	N47°40′58″ E16°50′37″	20.xi.2015
*Lepidocyrtus*	*curvicollis*	5	Bajót, Gerecse	cave	N47°43′22″ E18°34′31″	01.vi.2018
*Lepidocyrtus*	*cyaneus*	4	Tüskevár	grassland	N47°07′07″ E17°19′51″	03.xii.2015
*Lepidocyrtus*	*florae*	4	Hanság (loc. typ.)	alder forest	N47°40′47″ E17°07′53″	11.iv.2016
*Lepidocyrtus*	*isabelleae*	5	Nagykapornak	beech forest	N46°50′30″ E16°58′32″	15.vii.2017
*Lepidocyrtus*	*isabelleae*	4	Rum (loc. typ.)	riverine forest	N47°05′40″ E16°49′41″	09.viii.2016
*Lepidocyrtus*	*lanuginosus*	5	Sopron	urban park	N47°40′53″ E16°34′29″	02.iv.2016
*Lepidocyrtus*	*lignorum*	3	Sopron	grassland	N47°41′54″ E16°38′22″	24.x.2015
*Lepidocyrtus*	*lignorum*	1	Darvastó	oak forest	N46°58′56″ E17°22′12″	04.xii.2015
*Lepidocyrtus*	*lignorum*	4	Kerecsend	oak forest	N47°47′23″ E20°19′27″	01.x.2014
*Lepidocyrtus*	*mariani*	2	Nagykapornak	beech forest	N46°50′31″ E16°58′32″	01.vii.2014
*Lepidocyrtus*	*mariani*	3	Porva (loc. typ.)	alder forest	N47°18′42″ E17°47′30″	25.ix.2014
*Lepidocyrtus*	*nigrescens*	3	Kerecsend	grassland	N47°47′23″ E20°19′27″	01.x.2014
*Lepidocyrtus*	*nigrescens*	1	Darány	sedge	N45°58′03″ E17°32′18″	03.xi.2016
*Lepidocyrtus*	*paradoxus*	2	Fertőújlak	meadow	N47°40′58″ E16°50′37″	20.xi.2015
*Lepidocyrtus*	*paradoxus*	2	Bakonygyepes	we meadow	N47°08′29″ E17°33′37″	02.xii.2015
*Lepidocyrtus*	*peisonis*	4	Fertőrákos (loc. typ.)	reed-bed	N47°42′54″ E16°40′14″	25.iii.2017
*Lepidocyrtus*	*peisonis*	3	Darány	wet meadow	N45°59′14″ E17°33′23″	03.xi.2016
*Lepidocyrtus*	*serbicus*	5	Mátrafüred	beech	N47°53′26″ E19°56′37″	27.10.2016
*Lepidocyrtus*	*tomosvaryi*	5	Tüskevár (loc. typ.)	ash forest	N47°07′13″ E17°20′04″	02.xii.2015
*Lepidocyrtus*	*traseri*	3	Börzsöny (loc. typ.)	grassland	N47°53′55″ E18°51′08″	08.x.2014
*Lepidocyrtus*	*violaceus*	5	Csákvár	oak forest	N47°25′33″ E18°25′51″	13.ix.2016
*Orchesella*	*cincta*	1	Somlóvásárhely	grassland	N47° 8′43″ E17°22′35″	26.vi.2017
*Cyphoderus*	gr. *bidenticulati*	1	–	–	–	–

loc. typ.: Specimens collected from the species’ type locality.

**Table 2:** *Lepidocyrtus* species groups and related species examined.

Species Group/Species	Head Mac	Body Mac	Scales	Th. II	Abd. IV s	Abd. IV pse (dl-l)	Lateral Tuft	Dental Tubercle	Unguiculus
*L. lanuginosus-group*									
* L. cyaneus*	A_0_ A_2_ A_3_ M_2_ S_3_ Pa_5_	10/0101+2	–	n	–	–	–	–	A
* L. lanuginosus*	A_0_ A_2_ A_3_ M_2_ S_3_ Pa_5_	10/0101+2	–	n	–	–	–	–	A
*L. pallidus–serbicus* group									
* L. arrabonicus*	A_0_ A_2_ A_3_ Pa_5_	00/0101+2	–	n	–	–	–	–	A
* L. florae*	A_0_ A_2_ A_3_ S_3_ Pa_5_	00/0101+2	–	n	–	–	–	–	A
* L. isabelleae*	A_0_ A_2_ A_3_ M_2_ S_3_ Pa_5_	00/0101+2	–	n	–	–	–	–	A
* L. serbicus*	A_0_ A_2_ A_3_ M_2_ S_3_ Pa_5_	00/0101+2	–	n	–	–	–	–	A
* L. tomosvaryi*	A_0_ A_2_ A_3_ M_2_ S_3_ Pa_5_	00/0101+2	–	n	–	–	–	+	A
*L. lignorum* group									
* L. lignorum*	A_0_ A_2_ A_3_ Pa_5_	00/0101+3	+	p	–	–	–	–	A
* L. peisonis*	A_0_ A_2_ A_3_ Pa_5_	00/0101+3	+	p	–	–	+	–	T
* L. traseri*	A_0_ A_2_ A_3_ Pa_5_	00/0101+3	+	p	–	–	–	–	A
* L. violaceus*	A_0_ A_2_ A_3_ Pa_5_	00/0101+3	+	p	–	–	–	–	A
*L. curvicollis* group									
* L. curvicollis*	A_0_ A_2_ Pa_5_	00/0101+3	+	P	+	+	–	–	A
* L. mariani*	A_0_ A_2_ Pa_5_	00/0101+3	+	P	+	+	–	–	A
* L. nigrescens*	A_0_ A_2_ Pa_5_	00/0101+3	+	P	+	–	–	–	A
* L. paradoxus*	A_0_ A_2_ Pa_5_	00/0101+3	+	P	+	–	–	–	A

Head mac: dorsal cephalic macrochaetotaxy; Body mac: dorsal trunk macrochaetotaxy; Scales: Presence (+) or absence (–) of scales on antennae, legs (beyond coxae) and posterior face of manubrium; Th. II: Mesothorax projection–not projecting (n), slightly projecting (p), strongly projecting (P) over the head; Abd. IV s: Presence (+) or absence (–) of accessorial chaeta s on Abd. IV; Abd. IV pse (dl-l): Presence (+) or absence (–) of Abd. IV pseudopores in dorsolateral or lateral position; Dental tubercle: Presence (+) or absence (–) of dental tubercle on the basal part dens (dorsally); Unguiculus: shape of unguiculus–acuminate (A), truncate (T).

## Supplementary Materials

The following are available online at https://www.mdpi.com/2075-4450/11/5/302/s1, Table S1: GenBank accession numbers for nucleotide sequences generated for this study; Table S2: Pairwise distance matrix (uncorrected p-distances, %) for COII sequences; Table S3: Pairwise distance matrix (K2P, %) for COII sequences.
