# Supplementary material for: New Insight into the Systematics of European Lepidocyrtus (Collembola: Entomobryidae) Using Molecular and Morphological Data
Source: Insects. 2020 May 13;11(5):302. doi: 10.3390/insects11050302 (PMC7290404; doi:10.3390/insects11050302)
Supplement: Supplementary file 1 [file insects-11-00302-s001.pdf]

Supplementary material

Table S1. Specimen codes of the *Lepidocyrtus* species studied and GenBank accession numbers for nucleotide sequences generated for this study.

Species	Specimen code	GenBank ID	
		COII	EF1- α
L. arrabonicus	L030305	MT136169	
L. arrabonicus	L030306	MT136170	
L. arrabonicus	L042602	MT136171	
L. arrabonicus	L042604	MT136172	
L. cyaneus	L022302	MT136173	MT153249
L. cyaneus	L030102	MT136174	MT153250
L. cyaneus	L032910		MT153251
L. cyaneus	L032911	MT136175	MT153252
L. curvicollis	L180601	MT136176	MT153253
L. curvicollis	L180602	MT136177	MT153254
L. curvicollis	L180603	MT136178	MT153255
L. curvicollis	L180604	MT136179	
L. curvicollis	L180605	MT136180	
L. florae	L170311	MT136181	MT153256
L. florae	L170312	MT136182	
L. florae	L170314	MT136183	
L. florae	L170315	MT136184	
L. isabelleae	L041905	MT136185	MT153257
L. isabelleae	L041906	MT136186	MT153258
L. isabelleae	L041907	MT136187	MT153259
L. isabelleae	L041908	MT136188	MT153260
L. isabelleae	L041909	MT136189	
L. isabelleae	L180506	MT136190	MT153261
L. isabelleae	L180507	MT136191	MT153262
L. isabelleae	L180508	MT136192	
L. isabelleae	L180509	MT136193	
L. lanuginosus	L040215	MT136194	
L. lanuginosus	L042606	MT136195	MT153263
L. lanuginosus	L042608	MT136196	
L. lanuginosus	L042609	MT136197	
L. lanuginosus	L042610	MT136198	
L. lignorum	L030307	MT136199	
L. lignorum	L032903	MT136200	
L. lignorum	L032904	MT136201	
L. lignorum	L050306	MT136202	
L. lignorum	L050308	MT136203	
L. lignorum	L050315	MT136204	
L. lignorum	L051015	MT136205	
L. lignorum	L051016	MT136206	MT153264
L. mariani	L022305	MT136207	
L. mariani	L030105	MT136208	MT153265
L. mariani	L180501	MT136209	
L. mariani	L180502	MT136210	MT153266
L. mariani	L180504	MT136211	MT153267
L. nigrescens	L042613	MT136212	
L. nigrescens	L170601	MT136213	MT153268
L. nigrescens	L170604	MT136214	
L. nigrescens	L170605	MT136215	
L. paradoxus	L022303	MT136216	MT153269
L. paradoxus	L030103	MT136217	MT153270
L. paradoxus	L041913	MT136218	

L. paradoxus	L041914	MT136219	
L. peisonis	L170406	MT136220	MT153271
L. peisonis	L170408	MT136221	MT153272
L. peisonis	L170409		MT153273
L. peisonis	L170303	MT136222	MT153274
L. peisonis	L170304	MT136223	
L. peisonis	L170305	MT136224	MT153275
L. peisonis	L170611		MT153276
L. serbicus	L180521	MT136225	
L. serbicus	L180522		MT153277
L. serbicus	L180523	MT136226	MT153278
L. serbicus	L180524	MT136227	
L. serbicus	L180525	MT136228	MT153279
L. tomosvaryi	L031716	MT136229	MT153280
L. tomosvaryi	L031717	MT136230	MT153281
L. tomosvaryi	L032905	MT136231	MT153282
L. tomosvaryi	L032906	MT136232	MT153283
L. tomosvaryi	L032907	MT136233	MT153284
L. traseri	L030303	MT136234	MT153285
L. traseri	L031705	MT136235	
L. traseri	L041902	MT136236	
L. violaceus	L170316		MT153286
L. violaceus	L170317	MT136237	MT153287
L. violaceus	L170318	MT136238	
L. violaceus	L170319	MT136239	
L. violaceus	L170320	MT136240	
Ochesella cincta	L082913	MT136241	MT153288
Cyphoderus gr. *bidenticulati*	LP440–2	MF095527	MF095613

Table S2. Pairwise distance matrix (uncorrected p-distances, %) for COII sequences

	L. arr	*L. flo*	*L. cya*	*L. lan*	*L. isa*	*L. ser*	*L. tom*	*L. vio*	*L. lig 1*	*L. lig 2*	*L. tra*	*L. pei 1*	*L. pei 2*	*L. pei 3*	*L. nig*	*L. par*	*L. cur*	*L. mar*
L. arrabonicus (n=4)	1.0%																	
L. florae (n=4)	19.2%	2.9%																
L. cyaneus (n=3)	24.8%	24.9%	0.2%															
L. lanuginosus (n=5)	21.3%	23.9%	20.2%	0.0%														
L. isabelleae (n=9)	26.1%	25.5%	24.5%	25.3%	0.8%													
L. serbicus (n=4)	24.6%	25.7%	26.2%	23.9%	19.7%	3.5%												
L. tomosvaryi (n=5)	25.6%	26.9%	25.2%	26.9%	20.1%	21.7%	0.1%											
L. violaceus (n=4)	24.0%	27.1%	27.5%	23.7%	26.3%	24.1%	25.0%	0.0%										
L. lignorum 1 (n=4)	27.2%	26.5%	26.5%	26.0%	26.7%	26.3%	25.8%	20.1%	0.7%									
L. lignorum 2 (n=4)	24.6%	25.7%	25.2%	24.4%	25.8%	22.7%	25.9%	20.7%	14.7%	0.5%								
L. traseri (n=3)	24.5%	24.6%	24.7%	24.0%	25.5%	25.9%	25.5%	20.8%	19.6%	20.4%	1.9%							
L. peisonis 1 (n=2)	28.8%	29.1%	28.7%	26.4%	30.4%	28.0%	30.4%	25.0%	26.4%	24.0%	24.7%	0.0%						
L. peisonis 2 (n=1)	27.3%	28.7%	28.9%	29.3%	28.4%	28.7%	28.3%	25.4%	24.9%	25.0%	24.9%	25.2%	n.r.					
L. peisonis 3 (n=2)	27.1%	27.1%	27.2%	26.1%	28.5%	25.7%	27.9%	24.1%	23.4%	21.2%	22.9%	25.9%	26.6%	0.0%				
L. nigrescens (n=4)	24.4%	26.0%	26.8%	23.9%	28.3%	27.6%	28.2%	23.8%	24.9%	24.8%	23.5%	24.4%	26.5%	27.9%	3.7%			
L. paradoxus (n=4)	24.5%	26.2%	28.6%	26.9%	28.2%	28.5%	28.6%	24.3%	25.3%	25.8%	22.7%	23.8%	25.7%	29.3%	14.5%	2.0%		
L. curvicollis (n=5)	27.1%	29.1%	29.1%	24.9%	31.0%	27.5%	29.2%	25.5%	27.4%	25.6%	25.4%	26.7%	26.4%	27.1%	21.5%	24.3%	0.1%	
L. mariani (n=5)	28.0%	28.2%	28.2%	26.6%	29.4%	28.0%	28.5%	27.2%	29.7%	28.5%	26.0%	27.8%	29.7%	28.3%	26.6%	25.9%	24.1%	0.6%

Table S3. Pairwise distance matrix (K2P, %) for COII sequences

	L. arr	*L. flo*	*L. cya*	*L. lan*	*L. isa*	*L. ser*	*L. tom*	*L. vio*	*L. lig 1*	*L. lig 2*	*L. tra*	*L. pei 1*	*L. pei 2*	*L. pei 3*	*L. nig*	*L. par*	*L. cur*	*L. mar*
L. arrabonicus (n=4)	1.0%																	
L. florae (n=4)	22.5%	3.0%																
L. cyaneus (n=3)	30.7%	30.9%	0.2%															
L. lanuginosus (n=5)	25.3%	29.0%	23.8%	0.0%														
L. isabelleae (n=9)	32.5%	31.9%	30.3%	31.4%	0.8%													
L. serbicus (n=4)	30.3%	31.8%	32.8%	29.3%	23.3%	3.7%												
L. tomosvaryi (n=5)	31.6%	34.1%	31.3%	33.8%	23.7%	26.1%	0.1%											
L. violaceus (n=4)	29.1%	34.3%	35.2%	28.9%	33.4%	29.6%	30.9%	0.0%										
L. lignorum 1 (n=4)	34.3%	33.0%	33.1%	32.6%	33.8%	33.1%	32.3%	23.8%	0.7%									
L. lignorum 2 (n=4)	30.2%	31.8%	31.2%	29.9%	32.2%	27.5%	32.5%	24.6%	16.7%	0.5%								
L. traseri (n=3)	30.2%	30.2%	30.4%	29.4%	31.8%	32.5%	31.6%	25.0%	22.9%	24.1%	2.0%							
L. peisonis 1 (n=2)	36.8%	37.3%	36.9%	33.2%	39.5%	35.6%	39.7%	30.9%	33.2%	29.6%	30.5%	0.0%						
L. peisonis 2 (n=1)	34.3%	36.9%	37.6%	38.3%	36.1%	36.7%	36.0%	31.8%	31.0%	31.1%	30.9%	31.0%	n.r.					
L. peisonis 3 (n=2)	34.0%	34.1%	34.2%	32.6%	37.1%	32.1%	35.6%	29.8%	28.6%	25.3%	27.9%	32.5%	34.0%	0.0%				
L. nigrescens (n=4)	29.9%	32.3%	33.5%	28.8%	36.0%	34.9%	35.7%	29.0%	30.7%	30.4%	28.6%	29.8%	33.0%	35.6%	3.9%			
L. paradoxus (n=4)	29.8%	32.5%	36.5%	33.5%	35.8%	36.5%	36.5%	29.8%	31.3%	32.0%	27.4%	28.9%	31.7%	38.1%	16.4%	2.0%		
L. curvicollis (n=5)	34.2%	37.5%	37.7%	30.6%	40.8%	34.6%	37.7%	31.7%	34.7%	31.9%	31.6%	33.6%	32.9%	34.2%	25.7%	29.8%	0.1%	
L. mariani (n=5)	35.4%	35.9%	35.9%	33.3%	38.0%	35.7%	36.4%	34.2%	38.4%	36.8%	32.3%	35.6%	38.6%	36.7%	33.3%	32.2%	29.5%	0.6%
